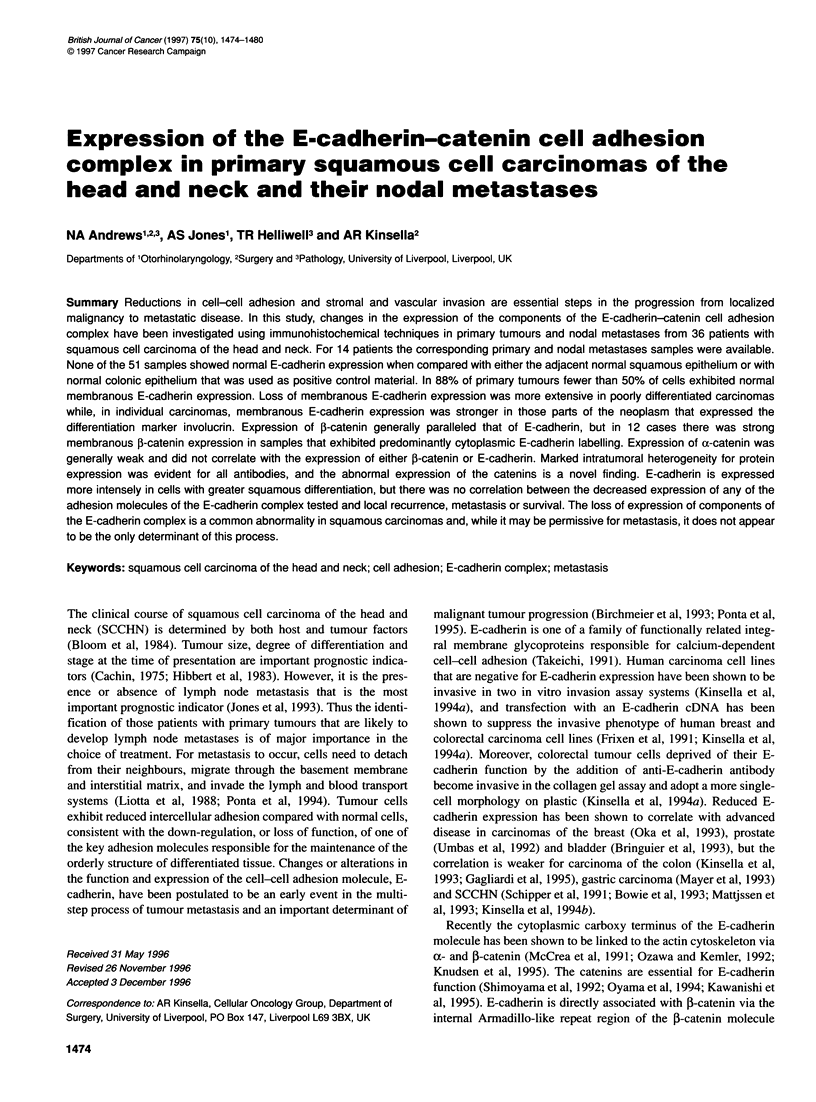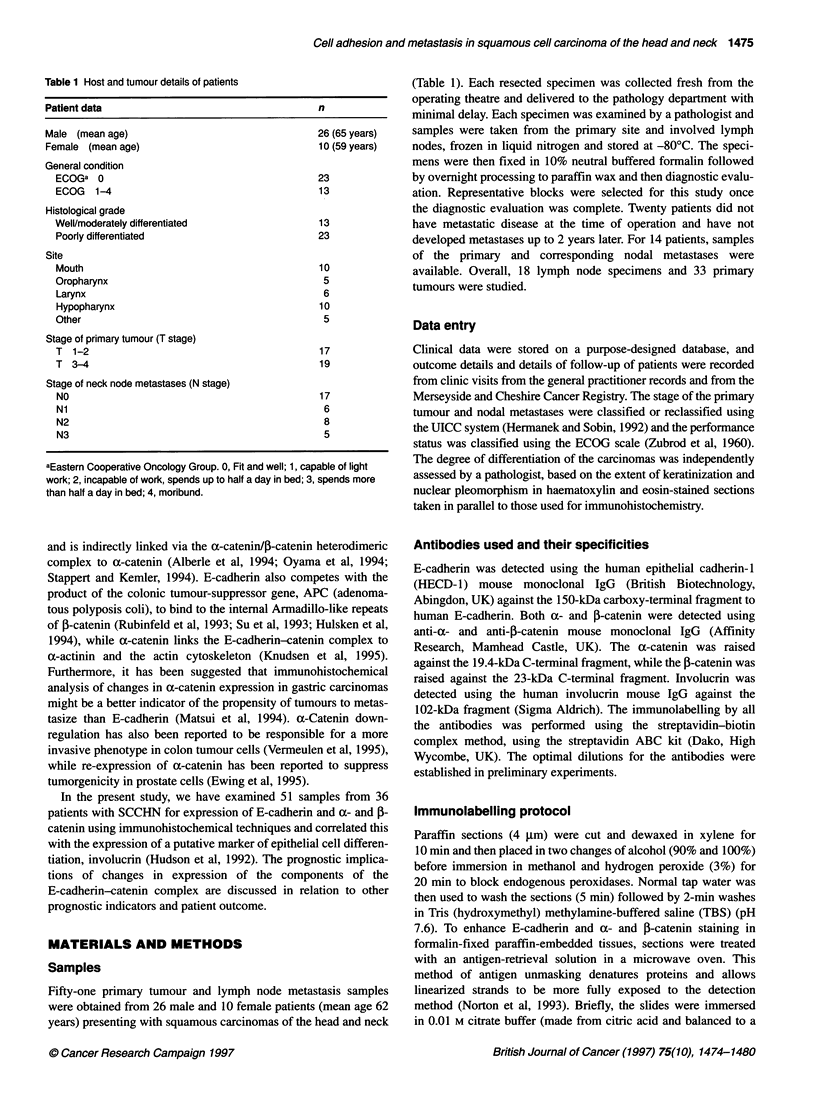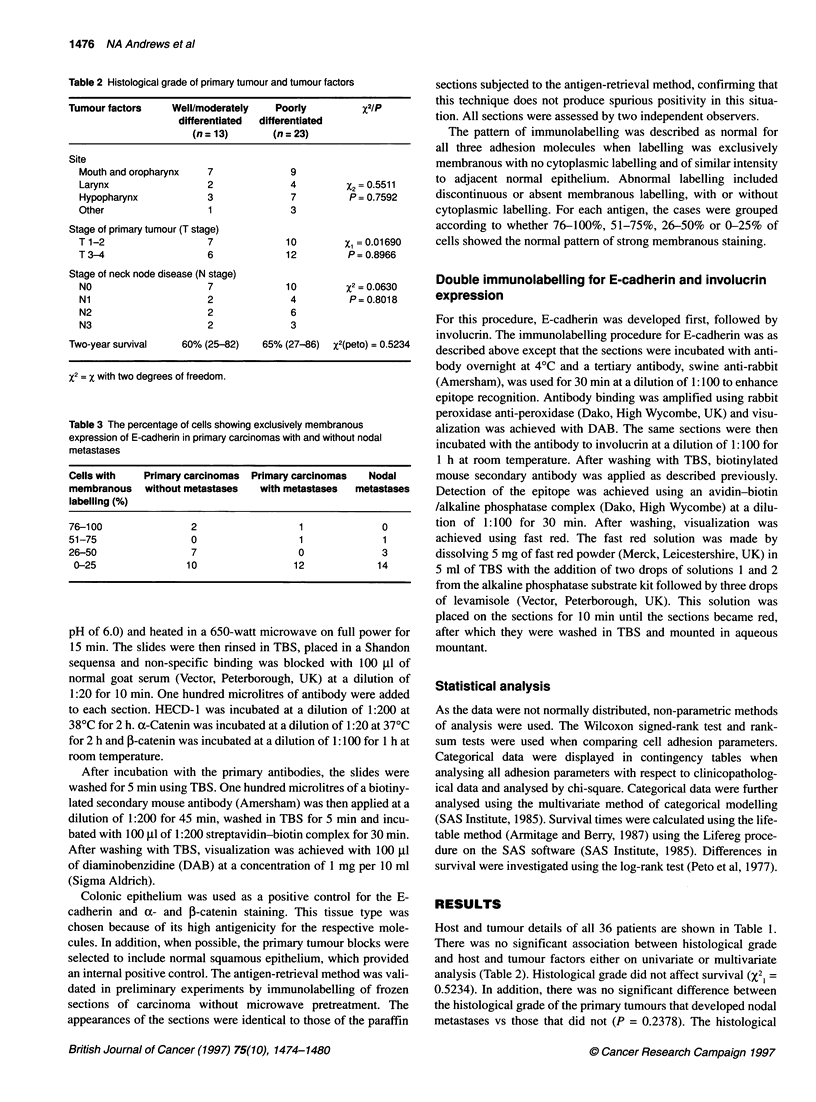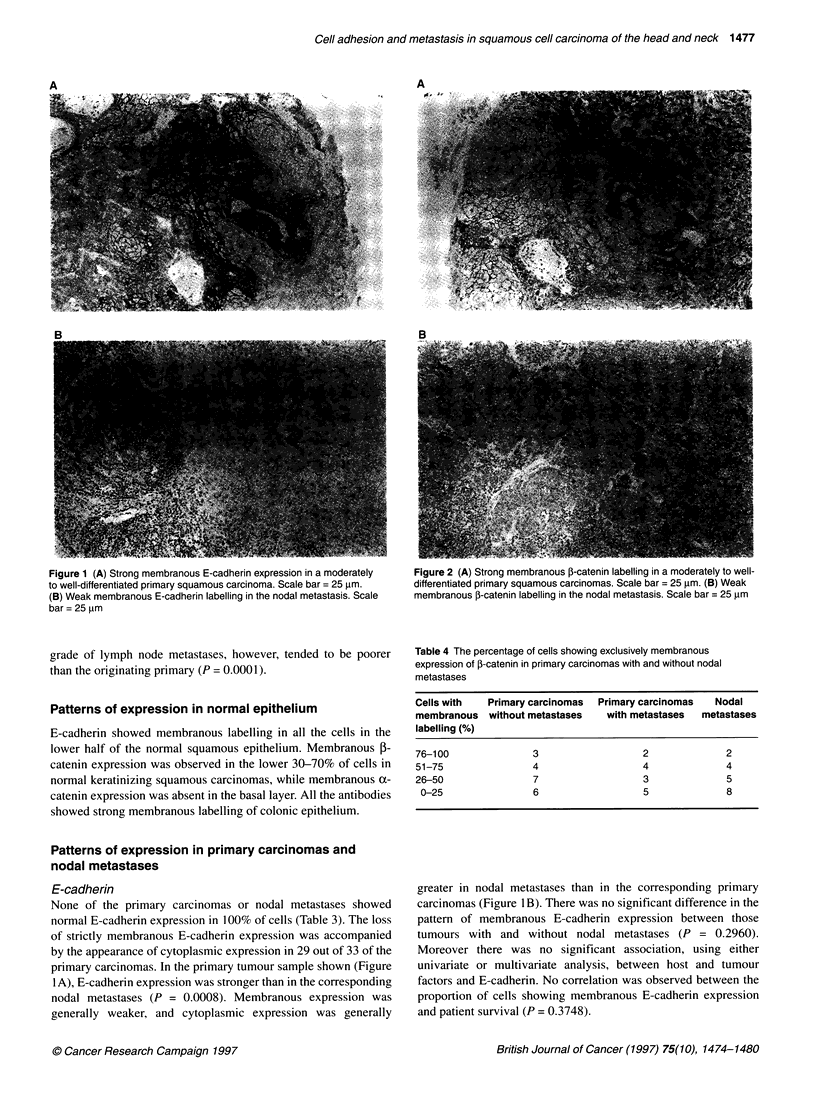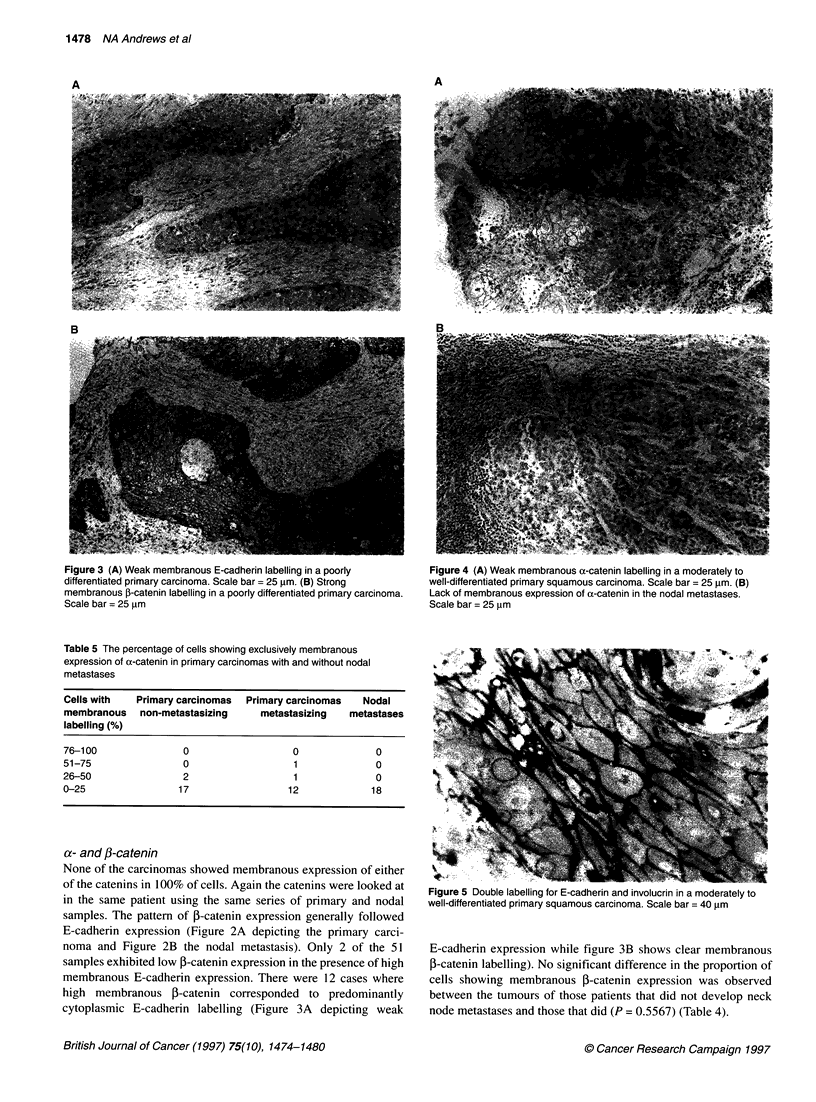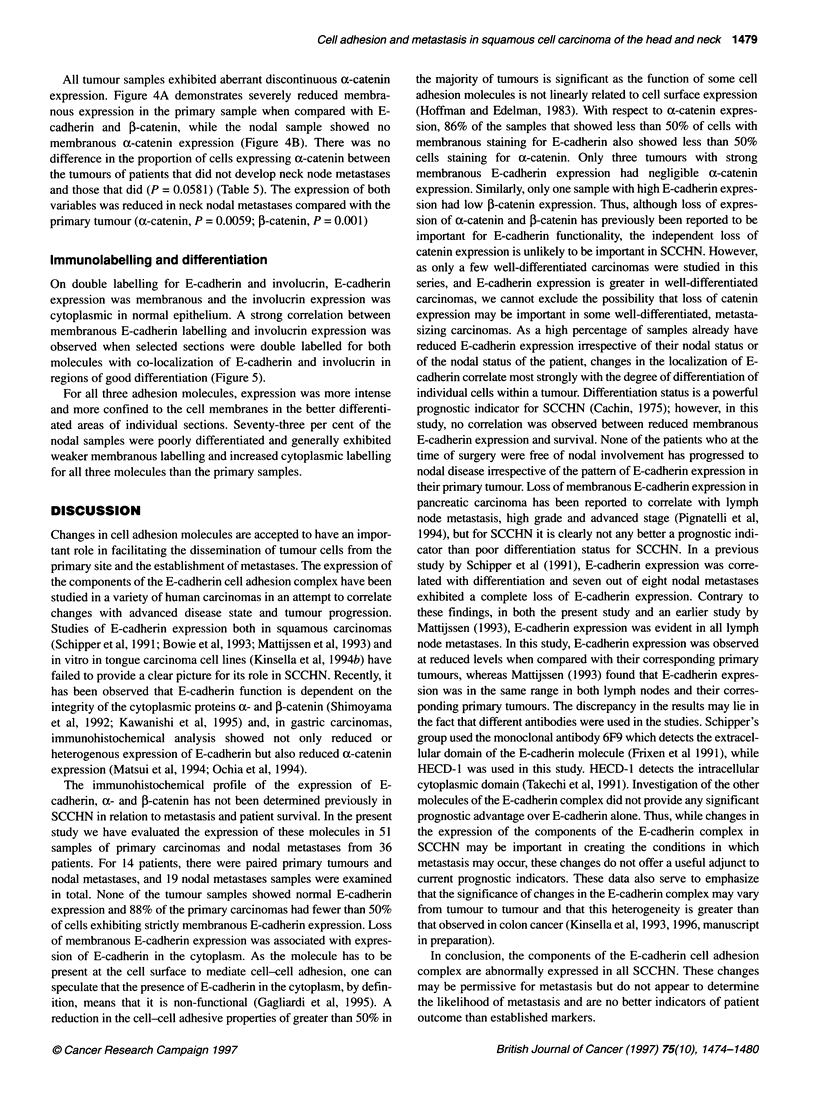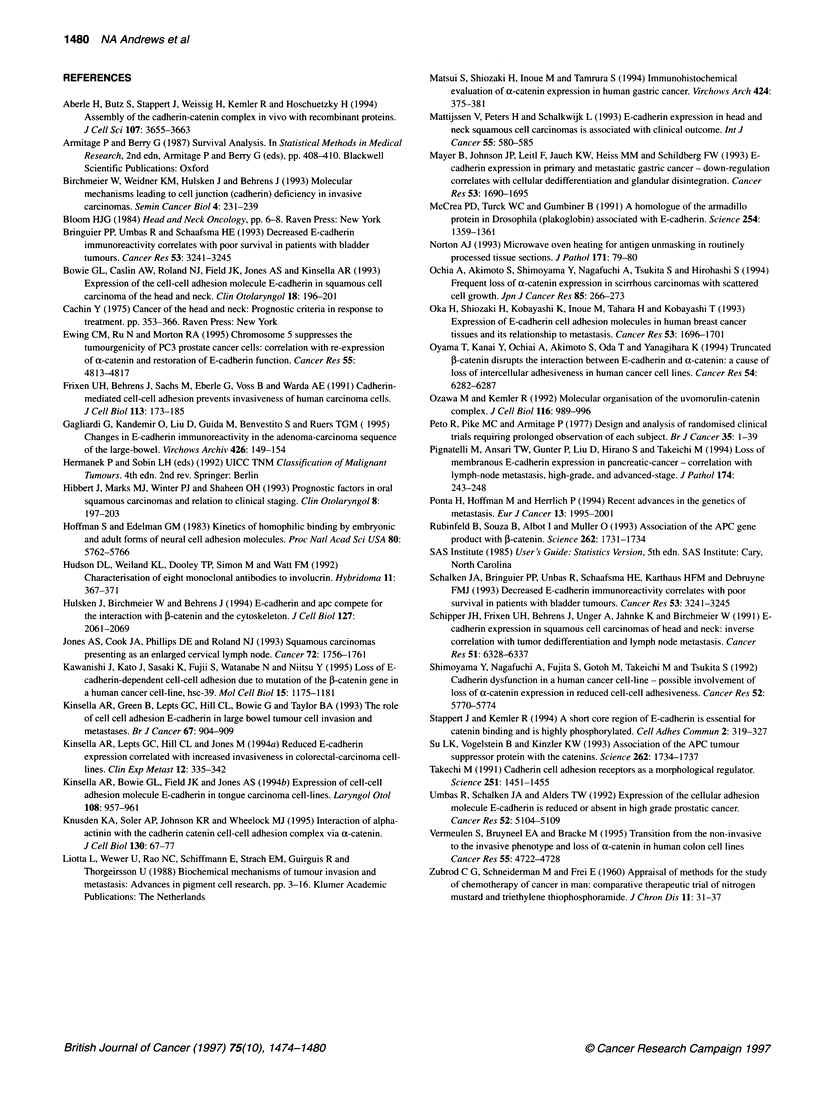# Expression of the E-cadherin-catenin cell adhesion complex in primary squamous cell carcinomas of the head and neck and their nodal metastases.

**DOI:** 10.1038/bjc.1997.252

**Published:** 1997

**Authors:** N. A. Andrews, A. S. Jones, T. R. Helliwell, A. R. Kinsella

**Affiliations:** Department of Otorhinolaryngology, University of Liverpool, UK.

## Abstract

**Images:**


British Journal of Cancer (1997) 75(10), 1474–1480
© 1997 Cancer Research Campaign

### Expression of the E-cadherin–catenin cell adhesion complex in primary squamous cell carcinomas of the head and neck and their nodal metastases

NA Andrews[1,2,3], AS Jones[1], TR Helliwell[3] and AR Kinsella[2]

Departments of [1]Otorhinolaryngology, [2]Surgery and [3]Pathology, University of Liverpool, Liverpool, UK

**Summary** Reductions in cell–cell adhesion and stromal and vascular invasion are essential steps in the progression from localized malignancy to metastatic disease. In this study, changes in the expression of the components of the E-cadherin–catenin cell adhesion complex have been investigated using immunohistochemical techniques in primary tumours and nodal metastases from 36 patients with squamous cell carcinoma of the head and neck. For 14 patients the corresponding primary and nodal metastases samples were available. None of the 51 samples showed normal E-cadherin expression when compared with either the adjacent normal squamous epithelium or with normal colonic epithelium that was used as positive control material. In 88% of primary tumours fewer than 50% of cells exhibited normal membranous E-cadherin expression. Loss of membranous E-cadherin expression was more extensive in poorly differentiated carcinomas while, in individual carcinomas, membranous E-cadherin expression was stronger in those parts of the neoplasm that expressed the differentiation marker involucrin. Expression of β-catenin generally paralleled that of E-cadherin, but in 12 cases there was strong membranous β-catenin expression in samples that exhibited predominantly cytoplasmic E-cadherin labelling. Expression of α-catenin was generally weak and did not correlate with the expression of either β-catenin or E-cadherin. Marked intratumoral heterogeneity for protein expression was evident for all antibodies, and the abnormal expression of the catenins is a novel finding. E-cadherin is expressed more intensely in cells with greater squamous differentiation, but there was no correlation between the decreased expression of any of the adhesion molecules of the E-cadherin complex tested and local recurrence, metastasis or survival. The loss of expression of components of the E-cadherin complex is a common abnormality in squamous carcinomas and, while it may be permissive for metastasis, it does not appear to be the only determinant of this process.

**Keywords:** squamous cell carcinoma of the head and neck; cell adhesion; E-cadherin complex; metastasis

The clinical course of squamous cell carcinoma of the head and neck (SCCHN) is determined by both host and tumour factors (Bloom et al, 1984). Tumour size, degree of differentiation and stage at the time of presentation are important prognostic indicators (Cachin, 1975; Hibbert et al, 1983). However, it is the presence or absence of lymph node metastasis that is the most important prognostic indicator (Jones et al, 1993). Thus the identification of those patients with primary tumours that are likely to develop lymph node metastases is of major importance in the choice of treatment. For metastasis to occur, cells need to detach from their neighbours, migrate through the basement membrane and interstitial matrix, and invade the lymph and blood transport systems (Liotta et al, 1988; Ponta et al, 1994). Tumour cells exhibit reduced intercellular adhesion compared with normal cells, consistent with the down-regulation, or loss of function, of one of the key adhesion molecules responsible for the maintenance of the orderly structure of differentiated tissue. Changes or alterations in the function and expression of the cell–cell adhesion molecule, E-cadherin, have been postulated to be an early event in the multistep process of tumour metastasis and an important determinant of malignant tumour progression (Birchmeier et al, 1993; Ponta et al, 1995). E-cadherin is one of a family of functionally related integral membrane glycoproteins responsible for calcium-dependent cell–cell adhesion (Takeichi, 1991). Human carcinoma cell lines that are negative for E-cadherin expression have been shown to be invasive in two in vitro invasion assay systems (Kinsella et al, 1994*a*), and transfection with an E-cadherin cDNA has been shown to suppress the invasive phenotype of human breast and colorectal carcinoma cell lines (Frixen et al, 1991; Kinsella et al, 1994*a*). Moreover, colorectal tumour cells deprived of their E-cadherin function by the addition of anti-E-cadherin antibody become invasive in the collagen gel assay and adopt a more single-cell morphology on plastic (Kinsella et al, 1994*a*). Reduced E-cadherin expression has been shown to correlate with advanced disease in carcinomas of the breast (Oka et al, 1993), prostate (Umbas et al, 1992) and bladder (Bringuier et al, 1993), but the correlation is weaker for carcinoma of the colon (Kinsella et al, 1993; Gagliardi et al, 1995), gastric carcinoma (Mayer et al, 1993) and SCCHN (Schipper et al, 1991; Bowie et al, 1993; Mattjssen et al, 1993; Kinsella et al, 1994*b*).

Recently the cytoplasmic carboxy terminus of the E-cadherin molecule has been shown to be linked to the actin cytoskeleton via α- and β-catenin (McCrea et al, 1991; Ozawa and Kemler, 1992; Knudsen et al, 1995). The catenins are essential for E-cadherin function (Shimoyama et al, 1992; Oyama et al, 1994; Kawanishi et al, 1995). E-cadherin is directly associated with β-catenin via the internal Armadillo-like repeat region of the β-catenin molecule

*Received 31 May 1996*
*Revised 26 November 1996*
*Accepted 3 December 1996*

*Correspondence to:* AR Kinsella, Cellular Oncology Group, Department of Surgery, University of Liverpool, PO Box 147, Liverpool L69 3BX, UK

**Table 1** Host and tumour details of patients

| Patient data | *n* |
|---|---|
| Male (mean age) | 26 (65 years) |
| Female (mean age) | 10 (59 years) |
| General condition | |
| ECOG[a] 0 | 23 |
| ECOG 1–4 | 13 |
| Histological grade | |
| Well/moderately differentiated | 13 |
| Poorly differentiated | 23 |
| Site | |
| Mouth | 10 |
| Oropharynx | 5 |
| Larynx | 6 |
| Hypopharynx | 10 |
| Other | 5 |
| Stage of primary tumour (T stage) | |
| T 1–2 | 17 |
| T 3–4 | 19 |
| Stage of neck node metastases (N stage) | |
| N0 | 17 |
| N1 | 6 |
| N2 | 8 |
| N3 | 5 |

[a]Eastern Cooperative Oncology Group. 0, Fit and well; 1, capable of light work; 2, incapable of work, spends up to half a day in bed; 3, spends more than half a day in bed; 4, moribund.

and is indirectly linked via the α-catenin/β-catenin heterodimeric complex to α-catenin (Alberle et al, 1994; Oyama et al, 1994; Stappert and Kemler, 1994). E-cadherin also competes with the product of the colonic tumour-suppressor gene, APC (adenomatous polyposis coli), to bind to the internal Armadillo-like repeats of β-catenin (Rubinfeld et al, 1993; Su et al, 1993; Hulsken et al, 1994), while α-catenin links the E-cadherin–catenin complex to α-actinin and the actin cytoskeleton (Knudsen et al, 1995). Furthermore, it has been suggested that immunohistochemical analysis of changes in α-catenin expression in gastric carcinomas might be a better indicator of the propensity of tumours to metastasize than E-cadherin (Matsui et al, 1994). α-Catenin down-regulation has also been reported to be responsible for a more invasive phenotype in colon tumour cells (Vermeulen et al, 1995), while re-expression of α-catenin has been reported to suppress tumorgenicity in prostate cells (Ewing et al, 1995).

In the present study, we have examined 51 samples from 36 patients with SCCHN for expression of E-cadherin and α- and β-catenin using immunohistochemical techniques and correlated this with the expression of a putative marker of epithelial cell differentiation, involucrin (Hudson et al, 1992). The prognostic implications of changes in expression of the components of the E-cadherin–catenin complex are discussed in relation to other prognostic indicators and patient outcome.

### MATERIALS AND METHODS

### Samples

Fifty-one primary tumour and lymph node metastasis samples were obtained from 26 male and 10 female patients (mean age 62 years) presenting with squamous carcinomas of the head and neck (Table 1). Each resected specimen was collected fresh from the operating theatre and delivered to the pathology department with minimal delay. Each specimen was examined by a pathologist and samples were taken from the primary site and involved lymph nodes, frozen in liquid nitrogen and stored at –80°C. The specimens were then fixed in 10% neutral buffered formalin followed by overnight processing to paraffin wax and then diagnostic evaluation. Representative blocks were selected for this study once the diagnostic evaluation was complete. Twenty patients did not have metastatic disease at the time of operation and have not developed metastases up to 2 years later. For 14 patients, samples of the primary and corresponding nodal metastases were available. Overall, 18 lymph node specimens and 33 primary tumours were studied.

### Data entry

Clinical data were stored on a purpose-designed database, and outcome details and details of follow-up of patients were recorded from clinic visits from the general practitioner records and from the Merseyside and Cheshire Cancer Registry. The stage of the primary tumour and nodal metastases were classified or reclassified using the UICC system (Hermanek and Sobin, 1992) and the performance status was classified using the ECOG scale (Zubrod et al, 1960). The degree of differentiation of the carcinomas was independently assessed by a pathologist, based on the extent of keratinization and nuclear pleomorphism in haematoxylin and eosin-stained sections taken in parallel to those used for immunohistochemistry.

### Antibodies used and their specificities

E-cadherin was detected using the human epithelial cadherin-1 (HECD-1) mouse monoclonal IgG (British Biotechnology, Abingdon, UK) against the 150-kDa carboxy-terminal fragment to human E-cadherin. Both α- and β-catenin were detected using anti-α- and anti-β-catenin mouse monoclonal IgG (Affinity Research, Mamhead Castle, UK). The α-catenin was raised against the 19.4-kDa C-terminal fragment, while the β-catenin was raised against the 23-kDa C-terminal fragment. Involucrin was detected using the human involucrin mouse IgG against the 102-kDa fragment (Sigma Aldrich). The immunolabelling by all the antibodies was performed using the streptavidin–biotin complex method, using the streptavidin ABC kit (Dako, High Wycombe, UK). The optimal dilutions for the antibodies were established in preliminary experiments.

### Immunolabelling protocol

Paraffin sections (4 μm) were cut and dewaxed in xylene for 10 min and then placed in two changes of alcohol (90% and 100%) before immersion in methanol and hydrogen peroxide (3%) for 20 min to block endogenous peroxidases. Normal tap water was then used to wash the sections (5 min) followed by 2-min washes in Tris (hydroxymethyl) methylamine-buffered saline (TBS) (pH 7.6). To enhance E-cadherin and α- and β-catenin staining in formalin-fixed paraffin-embedded tissues, sections were treated with an antigen-retrieval solution in a microwave oven. This method of antigen unmasking denatures proteins and allows linearized strands to be more fully exposed to the detection method (Norton et al, 1993). Briefly, the slides were immersed in 0.01 M citrate buffer (made from citric acid and balanced to a

© Cancer Research Campaign 1997 

Table 2 Histological grade of primary tumour and tumour factors

| Tumour factors | Well/moderately differentiated ($n$ = 13) | Poorly differentiated ($n$ = 23) | $\chi^2/P$ |
|---|---|---|---|
| Site | | | |
| Mouth and oropharynx | 7 | 9 | |
| Larynx | 2 | 4 | $\chi_2$ = 0.5511 |
| Hypopharynx | 3 | 7 | $P$ = 0.7592 |
| Other | 1 | 3 | |
| Stage of primary tumour (T stage) | | | |
| T 1–2 | 7 | 10 | $\chi_1$ = 0.01690 |
| T 3–4 | 6 | 12 | $P$ = 0.8966 |
| Stage of neck node disease (N stage) | | | |
| N0 | 7 | 10 | $\chi^2$ = 0.0630 |
| N1 | 2 | 4 | $P$ = 0.8018 |
| N2 | 2 | 6 | |
| N3 | 2 | 3 | |
| Two-year survival | 60% (25–82) | 65% (27–86) | $\chi^2$(peto) = 0.5234 |

$\chi^2 = \chi$ with two degrees of freedom.

Table 3 The percentage of cells showing exclusively membranous expression of E-cadherin in primary carcinomas with and without nodal metastases

| Cells with membranous labelling (%) | Primary carcinomas without metastases | Primary carcinomas with metastases | Nodal metastases |
|---|---|---|---|
| 76–100 | 2 | 1 | 0 |
| 51–75 | 0 | 1 | 1 |
| 26–50 | 7 | 0 | 3 |
| 0–25 | 10 | 12 | 14 |

pH of 6.0) and heated in a 650-watt microwave on full power for 15 min. The slides were then rinsed in TBS, placed in a Shandon sequensa and non-specific binding was blocked with 100 μl of normal goat serum (Vector, Peterborough, UK) at a dilution of 1:20 for 10 min. One hundred microlitres of antibody were added to each section. HECD-1 was incubated at a dilution of 1:200 at 38°C for 2 h. α-Catenin was incubated at a dilution of 1:20 at 37°C for 2 h and β-catenin was incubated at a dilution of 1:100 for 1 h at room temperature.

After incubation with the primary antibodies, the slides were washed for 5 min using TBS. One hundred microlitres of a biotinylated secondary mouse antibody (Amersham) was then applied at a dilution of 1:200 for 45 min, washed in TBS for 5 min and incubated with 100 μl of 1:200 streptavidin–biotin complex for 30 min. After washing with TBS, visualization was achieved with 100 μl of diaminobenzidine (DAB) at a concentration of 1 mg per 10 ml (Sigma Aldrich).

Colonic epithelium was used as a positive control for the E-cadherin and α- and β-catenin staining. This tissue type was chosen because of its high antigenicity for the respective molecules. In addition, when possible, the primary tumour blocks were selected to include normal squamous epithelium, which provided an internal positive control. The antigen-retrieval method was validated in preliminary experiments by immunolabelling of frozen sections of carcinoma without microwave pretreatment. The appearances of the sections were identical to those of the paraffin sections subjected to the antigen-retrieval method, confirming that this technique does not produce spurious positivity in this situation. All sections were assessed by two independent observers.

The pattern of immunolabelling was described as normal for all three adhesion molecules when labelling was exclusively membranous with no cytoplasmic labelling and of similar intensity to adjacent normal epithelium. Abnormal labelling included discontinuous or absent membranous labelling, with or without cytoplasmic labelling. For each antigen, the cases were grouped according to whether 76–100%, 51–75%, 26–50% or 0–25% of cells showed the normal pattern of strong membranous staining.

### Double immunolabelling for E-cadherin and involucrin expression

For this procedure, E-cadherin was developed first, followed by involucrin. The immunolabelling procedure for E-cadherin was as described above except that the sections were incubated with antibody overnight at 4°C and a tertiary antibody, swine anti-rabbit (Amersham), was used for 30 min at a dilution of 1:100 to enhance epitope recognition. Antibody binding was amplified using rabbit peroxidase anti-peroxidase (Dako, High Wycombe, UK) and visualization was achieved with DAB. The same sections were then incubated with the antibody to involucrin at a dilution of 1:100 for 1 h at room temperature. After washing with TBS, biotinylated mouse secondary antibody was applied as described previously. Detection of the epitope was achieved using an avidin–biotin /alkaline phosphatase complex (Dako, High Wycombe) at a dilution of 1:100 for 30 min. After washing, visualization was achieved using fast red. The fast red solution was made by dissolving 5 mg of fast red powder (Merck, Leicestershire, UK) in 5 ml of TBS with the addition of two drops of solutions 1 and 2 from the alkaline phosphatase substrate kit followed by three drops of levamisole (Vector, Peterborough, UK). This solution was placed on the sections for 10 min until the sections became red, after which they were washed in TBS and mounted in aqueous mountant.

### Statistical analysis

As the data were not normally distributed, non-parametric methods of analysis were used. The Wilcoxon signed-rank test and rank-sum tests were used when comparing cell adhesion parameters. Categorical data were displayed in contingency tables when analysing all adhesion parameters with respect to clinicopathological data and analysed by chi-square. Categorical data were further analysed using the multivariate method of categorical modelling (SAS Institute, 1985). Survival times were calculated using the life-table method (Armitage and Berry, 1987) using the Lifereg procedure on the SAS software (SAS Institute, 1985). Differences in survival were investigated using the log-rank test (Peto et al, 1977).

### RESULTS

Host and tumour details of all 36 patients are shown in Table 1. There was no significant association between histological grade and host and tumour factors either on univariate or multivariate analysis (Table 2). Histological grade did not affect survival ($\chi^2_1$ = 0.5234). In addition, there was no significant difference between the histological grade of the primary tumours that developed nodal metastases vs those that did not ($P$ = 0.2378). The histological

© Cancer Research Campaign 1997

**Figure 1** (**A**) Strong membranous E-cadherin expression in a moderately to well-differentiated primary squamous carcinoma. Scale bar = 25 μm. (**B**) Weak membranous E-cadherin labelling in the nodal metastasis. Scale bar = 25 μm

**Figure 2** (**A**) Strong membranous β-catenin labelling in a moderately to well-differentiated primary squamous carcinomas. Scale bar = 25 μm. (**B**) Weak membranous β-catenin labelling in the nodal metastasis. Scale bar = 25 μm grade of lymph node metastases, however, tended to be poorer than the originating primary ($P$ = 0.0001).

### Patterns of expression in normal epithelium

E-cadherin showed membranous labelling in all the cells in the lower half of the normal squamous epithelium. Membranous β-catenin expression was observed in the lower 30–70% of cells in normal keratinizing squamous carcinomas, while membranous α-catenin expression was absent in the basal layer. All the antibodies showed strong membranous labelling of colonic epithelium.

### Patterns of expression in primary carcinomas and nodal metastases

### *E-cadherin*

None of the primary carcinomas or nodal metastases showed normal E-cadherin expression in 100% of cells (Table 3). The loss of strictly membranous E-cadherin expression was accompanied by the appearance of cytoplasmic expression in 29 out of 33 of the primary carcinomas. In the primary tumour sample shown (Figure 1A), E-cadherin expression was stronger than in the corresponding nodal metastases ($P$ = 0.0008). Membranous expression was generally weaker, and cytoplasmic expression was generally greater in nodal metastases than in the corresponding primary carcinomas (Figure 1B). There was no significant difference in the pattern of membranous E-cadherin expression between those tumours with and without nodal metastases ($P$ = 0.2960). Moreover there was no significant association, using either univariate or multivariate analysis, between host and tumour factors and E-cadherin. No correlation was observed between the proportion of cells showing membranous E-cadherin expression and patient survival ($P$ = 0.3748).

**Table 4** The percentage of cells showing exclusively membranous expression of β-catenin in primary carcinomas with and without nodal metastases

| Cells with membranous labelling (%) | Primary carcinomas without metastases | Primary carcinomas with metastases | Nodal metastases |
|---|---|---|---|
| 76–100 | 3 | 2 | 2 |
| 51–75 | 4 | 4 | 4 |
| 26–50 | 7 | 3 | 5 |
| 0–25 | 6 | 5 | 8 |

© Cancer Research Campaign 1997 

**Figure 3** (**A**) Weak membranous E-cadherin labelling in a poorly differentiated primary carcinoma. Scale bar = 25 μm. (**B**) Strong membranous β-catenin labelling in a poorly differentiated primary carcinoma. Scale bar = 25 μm

**Figure 4** (**A**) Weak membranous α-catenin labelling in a moderately to well-differentiated primary squamous carcinoma. Scale bar = 25 μm. (**B**) Lack of membranous expression of α-catenin in the nodal metastases. Scale bar = 25 μm

**Table 5** The percentage of cells showing exclusively membranous expression of α-catenin in primary carcinomas with and without nodal metastases

| Cells with membranous labelling (%) | Primary carcinomas non-metastasizing | Primary carcinomas metastasizing | Nodal metastases |
|---|---|---|---|
| 76–100 | 0 | 0 | 0 |
| 51–75 | 0 | 1 | 0 |
| 26–50 | 2 | 1 | 0 |
| 0–25 | 17 | 12 | 18 |

### *α- and β-catenin*

None of the carcinomas showed membranous expression of either of the catenins in 100% of cells. Again the catenins were looked at in the same patient using the same series of primary and nodal samples. The pattern of β-catenin expression generally followed E-cadherin expression (Figure 2A depicting the primary carcinoma and Figure 2B the nodal metastasis). Only 2 of the 51 samples exhibited low β-catenin expression in the presence of high membranous E-cadherin expression. There were 12 cases where high membranous β-catenin corresponded to predominantly cytoplasmic E-cadherin labelling (Figure 3A depicting weak E-cadherin expression while figure 3B shows clear membranous β-catenin labelling). No significant difference in the proportion of cells showing membranous β-catenin expression was observed between the tumours of those patients that did not develop neck node metastases and those that did ($P$ = 0.5567) (Table 4).

**Figure 5** Double labelling for E-cadherin and involucrin in a moderately to well-differentiated primary squamous carcinoma. Scale bar = 40 μm

© Cancer Research Campaign 1997

All tumour samples exhibited aberrant discontinuous α-catenin expression. Figure 4A demonstrates severely reduced membranous expression in the primary sample when compared with E-cadherin and β-catenin, while the nodal sample showed no membranous α-catenin expression (Figure 4B). There was no difference in the proportion of cells expressing α-catenin between the tumours of patients that did not develop neck node metastases and those that did ($P = 0.0581$) (Table 5). The expression of both variables was reduced in neck nodal metastases compared with the primary tumour (α-catenin, $P = 0.0059$; β-catenin, $P = 0.001$)

### Immunolabelling and differentiation

On double labelling for E-cadherin and involucrin, E-cadherin expression was membranous and the involucrin expression was cytoplasmic in normal epithelium. A strong correlation between membranous E-cadherin labelling and involucrin expression was observed when selected sections were double labelled for both molecules with co-localization of E-cadherin and involucrin in regions of good differentiation (Figure 5).

For all three adhesion molecules, expression was more intense and more confined to the cell membranes in the better differentiated areas of individual sections. Seventy-three per cent of the nodal samples were poorly differentiated and generally exhibited weaker membranous labelling and increased cytoplasmic labelling for all three molecules than the primary samples.

### DISCUSSION

Changes in cell adhesion molecules are accepted to have an important role in facilitating the dissemination of tumour cells from the primary site and the establishment of metastases. The expression of the components of the E-cadherin cell adhesion complex have been studied in a variety of human carcinomas in an attempt to correlate changes with advanced disease state and tumour progression. Studies of E-cadherin expression both in squamous carcinomas (Schipper et al, 1991; Bowie et al, 1993; Mattijssen et al, 1993) and in vitro in tongue carcinoma cell lines (Kinsella et al, 1994*b*) have failed to provide a clear picture for its role in SCCHN. Recently, it has been observed that E-cadherin function is dependent on the integrity of the cytoplasmic proteins α- and β-catenin (Shimoyama et al, 1992; Kawanishi et al, 1995) and, in gastric carcinomas, immunohistochemical analysis showed not only reduced or heterogenous expression of E-cadherin but also reduced α-catenin expression (Matsui et al, 1994; Ochia et al, 1994).

The immunohistochemical profile of the expression of E-cadherin, α- and β-catenin has not been determined previously in SCCHN in relation to metastasis and patient survival. In the present study we have evaluated the expression of these molecules in 51 samples of primary carcinomas and nodal metastases from 36 patients. For 14 patients, there were paired primary tumours and nodal metastases, and 19 nodal metastases samples were examined in total. None of the tumour samples showed normal E-cadherin expression and 88% of the primary carcinomas had fewer than 50% of cells exhibiting strictly membranous E-cadherin expression. Loss of membranous E-cadherin expression was associated with expression of E-cadherin in the cytoplasm. As the molecule has to be present at the cell surface to mediate cell–cell adhesion, one can speculate that the presence of E-cadherin in the cytoplasm, by definition, means that it is non-functional (Gagliardi et al, 1995). A reduction in the cell–cell adhesive properties of greater than 50% in the majority of tumours is significant as the function of some cell adhesion molecules is not linearly related to cell surface expression (Hoffman and Edelman, 1983). With respect to α-catenin expression, 86% of the samples that showed less than 50% of cells with membranous staining for E-cadherin also showed less than 50% cells staining for α-catenin. Only three tumours with strong membranous E-cadherin expression had negligible α-catenin expression. Similarly, only one sample with high E-cadherin expression had low β-catenin expression. Thus, although loss of expression of α-catenin and β-catenin has previously been reported to be important for E-cadherin functionality, the independent loss of catenin expression is unlikely to be important in SCCHN. However, as only a few well-differentiated carcinomas were studied in this series, and E-cadherin expression is greater in well-differentiated carcinomas, we cannot exclude the possibility that loss of catenin expression may be important in some well-differentiated, metastasizing carcinomas. As a high percentage of samples already have reduced E-cadherin expression irrespective of their nodal status or of the nodal status of the patient, changes in the localization of E-cadherin correlate most strongly with the degree of differentiation of individual cells within a tumour. Differentiation status is a powerful prognostic indicator for SCCHN (Cachin, 1975); however, in this study, no correlation was observed between reduced membranous E-cadherin expression and survival. None of the patients who at the time of surgery were free of nodal involvement has progressed to nodal disease irrespective of the pattern of E-cadherin expression in their primary tumour. Loss of membranous E-cadherin expression in pancreatic carcinoma has been reported to correlate with lymph node metastasis, high grade and advanced stage (Pignatelli et al, 1994), but for SCCHN it is clearly not any better a prognostic indicator than poor differentiation status for SCCHN. In a previous study by Schipper et al (1991), E-cadherin expression was correlated with differentiation and seven out of eight nodal metastases exhibited a complete loss of E-cadherin expression. Contrary to these findings, in both the present study and an earlier study by Mattijssen (1993), E-cadherin expression was evident in all lymph node metastases. In this study, E-cadherin expression was observed at reduced levels when compared with their corresponding primary tumours, whereas Mattijssen (1993) found that E-cadherin expression was in the same range in both lymph nodes and their corresponding primary tumours. The discrepancy in the results may lie in the fact that different antibodies were used in the studies. Schipper's group used the monoclonal antibody 6F9 which detects the extracellular domain of the E-cadherin molecule (Frixen et al 1991), while HECD-1 was used in this study. HECD-1 detects the intracellular cytoplasmic domain (Takechi et al, 1991). Investigation of the other molecules of the E-cadherin complex did not provide any significant prognostic advantage over E-cadherin alone. Thus, while changes in the expression of the components of the E-cadherin complex in SCCHN may be important in creating the conditions in which metastasis may occur, these changes do not offer a useful adjunct to current prognostic indicators. These data also serve to emphasize that the significance of changes in the E-cadherin complex may vary from tumour to tumour and that this heterogeneity is greater than that observed in colon cancer (Kinsella et al, 1993, 1996, manuscript in preparation).

In conclusion, the components of the E-cadherin cell adhesion complex are abnormally expressed in all SCCHN. These changes may be permissive for metastasis but do not appear to determine the likelihood of metastasis and are no better indicators of patient outcome than established markers.

© Cancer Research Campaign 1997

### REFERENCES

Aberle H, Butz S, Stappert J, Weissig H, Kemler R and Hoschuetzky H (1994) Assembly of the cadherin-catenin complex in vivo with recombinant proteins. *J Cell Sci* **107**: 3655–3663

Armitage P and Berry G (1987) Survival Analysis. In *Statistical Methods in Medical Research*, 2nd edn, Armitage P and Berry G (eds), pp. 408–410. Blackwell Scientific Publications: Oxford

Birchmeier W, Weidner KM, Hulsken J and Behrens J (1993) Molecular mechanisms leading to cell junction (cadherin) deficiency in invasive carcinomas. *Semin Cancer Biol* **4**: 231–239

Bloom HJG (1984) *Head and Neck Oncology*, pp. 6–8. Raven Press: New York

Bringuier PP, Umbas R and Schaafsma HE (1993) Decreased E-cadherin immunoreactivity correlates with poor survival in patients with bladder tumours. *Cancer Res* **53**: 3241–3245

Bowie GL, Caslin AW, Roland NJ, Field JK, Jones AS and Kinsella AR (1993) Expression of the cell-cell adhesion molecule E-cadherin in squamous cell carcinoma of the head and neck. *Clin Otolaryngol* **18**: 196–201

Cachin Y (1975) Cancer of the head and neck: Prognostic criteria in response to treatment. pp. 353–366. Raven Press: New York

Ewing CM, Ru N and Morton RA (1995) Chromosome 5 suppresses the tumourgenicity of PC3 prostate cancer cells: correlation with re-expression of α-catenin and restoration of E-cadherin function. *Cancer Res* **55**: 4813–4817

Frixen UH, Behrens J, Sachs M, Eberle G, Voss B and Warda AE (1991) Cadherin-mediated cell-cell adhesion prevents invasiveness of human carcinoma cells. *J Cell Biol* **113**: 173–185

Gagliardi G, Kandemir O, Liu D, Guida M, Benvestito S and Ruers TGM ( 1995) Changes in E-cadherin immunoreactivity in the adenoma-carcinoma sequence of the large-bowel. *Virchows Archiv* **426**: 149–154

Hermanek P and Sobin LH (eds) (1992) UICC TNM *Classification of Malignant Tumours*. 4th edn. 2nd rev. Springer: Berlin

Hibbert J, Marks MJ, Winter PJ and Shaheen OH (1993) Prognostic factors in oral squamous carcinomas and relation to clinical staging. *Clin Otolaryngol* **8**: 197–203

Hoffman S and Edelman GM (1983) Kinetics of homophilic binding by embryonic and adult forms of neural cell adhesion molecules. *Proc Natl Acad Sci USA* **80**: 5762–5766

Hudson DL, Weiland KL, Dooley TP, Simon M and Watt FM (1992) Characterisation of eight monoclonal antibodies to involucrin. *Hybridoma* **11**: 367–371

Hulsken J, Birchmeier W and Behrens J (1994) E-cadherin and apc compete for the interaction with β-catenin and the cytoskeleton. *J Cell Biol* **127**: 2061–2069

Jones AS, Cook JA, Phillips DE and Roland NJ (1993) Squamous carcinomas presenting as an enlarged cervical lymph node. *Cancer* **72**: 1756–1761

Kawanishi J, Kato J, Sasaki K, Fujii S, Watanabe N and Niitsu Y (1995) Loss of E-cadherin-dependent cell-cell adhesion due to mutation of the β-catenin gene in a human cancer cell-line, hsc-39. *Mol Cell Biol* **15**: 1175–1181

Kinsella AR, Green B, Lepts GC, Hill CL, Bowie G and Taylor BA (1993) The role of cell cell adhesion E-cadherin in large bowel tumour cell invasion and metastases. *Br J Cancer* **67**: 904–909

Kinsella AR, Lepts GC, Hill CL and Jones M (1994*a*) Reduced E-cadherin expression correlated with increased invasiveness in colorectal-carcinoma cell-lines. *Clin Exp Metast* **12**: 335–342

Kinsella AR, Bowie GL, Field JK and Jones AS (1994*b*) Expression of cell-cell adhesion molecule E-cadherin in tongue carcinoma cell-lines. *Laryngol Otol* **108**: 957–961

Knusden KA, Soler AP, Johnson KR and Wheelock MJ (1995) Interaction of alpha-actinin with the cadherin catenin cell-cell adhesion complex via α-catenin. *J Cell Biol* **130**: 67–77

Liotta L, Wewer U, Rao NC, Schiffmann E, Strach EM, Guirguis R and Thorgeirsson U (1988) Biochemical mechanisms of tumour invasion and metastasis: Advances in pigment cell research, pp. 3–16. Klumer Academic Publications: The Netherlands

Matsui S, Shiozaki H, Inoue M and Tamrura S (1994) Immunohistochemical evaluation of α-catenin expression in human gastric cancer. *Virchows Arch* **424**: 375–381

Mattijssen V, Peters H and Schalkwijk L (1993) E-cadherin expression in head and neck squamous cell carcinomas is associated with clinical outcome. *Int J Cancer* **55**: 580–585

Mayer B, Johnson JP, Leitl F, Jauch KW, Heiss MM and Schildberg FW (1993) E-cadherin expression in primary and metastatic gastric cancer – down-regulation correlates with cellular dedifferentiation and glandular disintegration. *Cancer Res* **53**: 1690–1695

McCrea PD, Turck WC and Gumbiner B (1991) A homologue of the armadillo protein in Drosophila (plakoglobin) associated with E-cadherin. *Science* **254**: 1359–1361

Norton AJ (1993) Microwave oven heating for antigen unmasking in routinely processed tissue sections. *J Pathol* **171**: 79–80

Ochia A, Akimoto S, Shimoyama Y, Nagafuchi A, Tsukita S and Hirohashi S (1994) Frequent loss of α-catenin expression in scirrhous carcinomas with scattered cell growth. *Jpn J Cancer Res* **85**: 266–273

Oka H, Shiozaki H, Kobayashi K, Inoue M, Tahara H and Kobayashi T (1993) Expression of E-cadherin cell adhesion molecules in human breast cancer tissues and its relationship to metastasis. *Cancer Res* **53**: 1696–1701

Oyama T, Kanai Y, Ochiai A, Akimoto S, Oda T and Yanagihara K (1994) Truncated β-catenin disrupts the interaction between E-cadherin and α-catenin: a cause of loss of intercellular adhesiveness in human cancer cell lines. *Cancer Res* **54**: 6282–6287

Ozawa M and Kemler R (1992) Molecular organisation of the uvomorulin-catenin complex. *J Cell Biol* **116**: 989–996

Peto R, Pike MC and Armitage P (1977) Design and analysis of randomised clinical trials requiring prolonged observation of each subject. *Br J Cancer* **35**: 1–39

Pignatelli M, Ansari TW, Gunter P, Liu D, Hirano S and Takeichi M (1994) Loss of membranous E-cadherin expression in pancreatic-cancer – correlation with lymph-node metastasis, high-grade, and advanced-stage. *J Pathol* **174**: 243–248

Ponta H, Hoffman M and Herrlich P (1994) Recent advances in the genetics of metastasis. *Eur J Cancer* **13**: 1995–2001

Rubinfeld B, Souza B, Albot I and Muller O (1993) Association of the APC gene product with β-catenin. *Science* **262**: 1731–1734

SAS Institute (1985) *User's Guide: Statistics Version*, 5th edn. SAS Institute: Cary, North Carolina

Schalken JA, Bringuier PP, Unbas R, Schaafsma HE, Karthaus HFM and Debruyne FMJ (1993) Decreased E-cadherin immunoreactivity correlates with poor survival in patients with bladder tumours. *Cancer Res* **53**: 3241–3245

Schipper JH, Frixen UH, Behrens J, Unger A, Jahnke K and Birchmeier W (1991) E-cadherin expression in squamous cell carcinomas of head and neck: inverse correlation with tumor dedifferentiation and lymph node metastasis. *Cancer Res* **51**: 6328–6337

Shimoyama Y, Nagafuchi A, Fujita S, Gotoh M, Takeichi M and Tsukita S (1992) Cadherin dysfunction in a human cancer cell-line – possible involvement of loss of α-catenin expression in reduced cell-cell adhesiveness. *Cancer Res* **52**: 5770–5774

Stappert J and Kemler R (1994) A short core region of E-cadherin is essential for catenin binding and is highly phosphorylated. *Cell Adhes Commun* **2**: 319–327

Su LK, Vogelstein B and Kinzler KW (1993) Association of the APC tumour suppressor protein with the catenins. *Science* **262**: 1734–1737

Takechi M (1991) Cadherin cell adhesion receptors as a morphological regulator. *Science* **251**: 1451–1455

Umbas R, Schalken JA and Alders TW (1992) Expression of the cellular adhesion molecule E-cadherin is reduced or absent in high grade prostatic cancer. *Cancer Res* **52**: 5104–5109

Vermeulen S, Bruyneel EA and Bracke M (1995) Transition from the non-invasive to the invasive phenotype and loss of α-catenin in human colon cell lines *Cancer Res* **55**: 4722–4728

Zubrod C G, Schneiderman M and Frei E (1960) Appraisal of methods for the study of chemotherapy of cancer in man: comparative therapeutic trial of nitrogen mustard and triethylene thiophosphoramide. *J Chron Dis* **11**: 31–37

© Cancer Research Campaign 1997